# Supplementary material for: Unveiling the Bioactive Potential of the Invasive Jellyfish Phyllorhiza punctata Through Integrative Transcriptomic and Proteomic Analyses
Source: Biomolecules. 2025 Aug 4;15(8):1121. doi: 10.3390/biom15081121 (PMC12383608; doi:10.3390/biom15081121)
Supplement: Supplementary file 1 [file biomolecules-15-01121-s001.zip › Suplementary Material/FileS3/PEPFOLD4/XP_065068103_13697/clusters.html]

 PEP-FOLD clusters


Download archive of all models  here   

|  |  |  |
| --- | --- | --- |
| # | file | sOPEP |
| # cluster 1 - Size: 97 |  |  |
|  | XP\_065068103\_13697-model1 | -21.8798 |
|  | XP\_065068103\_13697-model1.1 | -21.8798 |
|  | XP\_065068103\_13697-model1.2 | -21.8392 |
|  | XP\_065068103\_13697-model1.3 | -21.7998 |
|  | XP\_065068103\_13697-model1.4 | -21.7776 |
|  | XP\_065068103\_13697-model1.5 | -21.767 |
|  | XP\_065068103\_13697-model1.6 | -21.7515 |
|  | XP\_065068103\_13697-model1.7 | -21.7291 |
|  | XP\_065068103\_13697-model1.8 | -21.7185 |
|  | XP\_065068103\_13697-model1.9 | -21.713 |
|  | XP\_065068103\_13697-model1.10 | -21.6797 |
|  | XP\_065068103\_13697-model1.11 | -21.6769 |
|  | XP\_065068103\_13697-model1.12 | -21.6729 |
|  | XP\_065068103\_13697-model1.13 | -21.6705 |
|  | XP\_065068103\_13697-model1.14 | -21.6678 |
|  | XP\_065068103\_13697-model1.15 | -21.6614 |
|  | XP\_065068103\_13697-model1.16 | -21.6573 |
|  | XP\_065068103\_13697-model1.17 | -21.6509 |
|  | XP\_065068103\_13697-model1.18 | -21.6496 |
|  | XP\_065068103\_13697-model1.19 | -21.6288 |
|  | XP\_065068103\_13697-model1.20 | -21.6288 |
|  | XP\_065068103\_13697-model1.21 | -21.6267 |
|  | XP\_065068103\_13697-model1.22 | -21.6261 |
|  | XP\_065068103\_13697-model1.23 | -21.6261 |
|  | XP\_065068103\_13697-model1.24 | -21.6216 |
|  | XP\_065068103\_13697-model1.25 | -21.6187 |
|  | XP\_065068103\_13697-model1.26 | -21.6187 |
|  | XP\_065068103\_13697-model1.27 | -21.6175 |
|  | XP\_065068103\_13697-model1.28 | -21.6161 |
|  | XP\_065068103\_13697-model1.29 | -21.6131 |
|  | XP\_065068103\_13697-model1.30 | -21.6095 |
|  | XP\_065068103\_13697-model1.31 | -21.6042 |
|  | XP\_065068103\_13697-model1.32 | -21.5964 |
|  | XP\_065068103\_13697-model1.33 | -21.5872 |
|  | XP\_065068103\_13697-model1.34 | -21.5809 |
|  | XP\_065068103\_13697-model1.35 | -21.5791 |
|  | XP\_065068103\_13697-model1.36 | -21.563 |
|  | XP\_065068103\_13697-model1.37 | -21.5625 |
|  | XP\_065068103\_13697-model1.38 | -21.5566 |
|  | XP\_065068103\_13697-model1.39 | -21.5566 |
|  | XP\_065068103\_13697-model1.40 | -21.5566 |
|  | XP\_065068103\_13697-model1.41 | -21.5566 |
|  | XP\_065068103\_13697-model1.42 | -21.547 |
|  | XP\_065068103\_13697-model1.43 | -21.5414 |
|  | XP\_065068103\_13697-model1.44 | -21.5381 |
|  | XP\_065068103\_13697-model1.45 | -21.5339 |
|  | XP\_065068103\_13697-model1.46 | -21.5272 |
|  | XP\_065068103\_13697-model1.47 | -21.5207 |
|  | XP\_065068103\_13697-model1.48 | -21.5207 |
|  | XP\_065068103\_13697-model1.49 | -21.5164 |
|  | XP\_065068103\_13697-model1.50 | -21.5158 |
|  | XP\_065068103\_13697-model1.51 | -21.5154 |
|  | XP\_065068103\_13697-model1.52 | -21.5139 |
|  | XP\_065068103\_13697-model1.53 | -21.5134 |
|  | XP\_065068103\_13697-model1.54 | -21.5119 |
|  | XP\_065068103\_13697-model1.55 | -21.5075 |
|  | XP\_065068103\_13697-model1.56 | -21.5046 |
|  | XP\_065068103\_13697-model1.57 | -21.5042 |
|  | XP\_065068103\_13697-model1.58 | -21.5038 |
|  | XP\_065068103\_13697-model1.59 | -21.5022 |
|  | XP\_065068103\_13697-model1.60 | -21.5017 |
|  | XP\_065068103\_13697-model1.61 | -21.4976 |
|  | XP\_065068103\_13697-model1.62 | -21.4956 |
|  | XP\_065068103\_13697-model1.63 | -21.4937 |
|  | XP\_065068103\_13697-model1.64 | -21.4909 |
|  | XP\_065068103\_13697-model1.65 | -21.4865 |
|  | XP\_065068103\_13697-model1.66 | -21.4813 |
|  | XP\_065068103\_13697-model1.67 | -21.4699 |
|  | XP\_065068103\_13697-model1.68 | -21.4698 |
|  | XP\_065068103\_13697-model1.69 | -21.4676 |
|  | XP\_065068103\_13697-model1.70 | -21.4669 |
|  | XP\_065068103\_13697-model1.71 | -21.4639 |
|  | XP\_065068103\_13697-model1.72 | -21.4511 |
|  | XP\_065068103\_13697-model1.73 | -21.4492 |
|  | XP\_065068103\_13697-model1.74 | -21.4492 |
|  | XP\_065068103\_13697-model1.75 | -21.4481 |
|  | XP\_065068103\_13697-model1.76 | -21.4481 |
|  | XP\_065068103\_13697-model1.77 | -21.4481 |
|  | XP\_065068103\_13697-model1.78 | -21.4283 |
|  | XP\_065068103\_13697-model1.79 | -21.4283 |
|  | XP\_065068103\_13697-model1.80 | -21.427 |
|  | XP\_065068103\_13697-model1.81 | -21.4239 |
|  | XP\_065068103\_13697-model1.82 | -21.4167 |
|  | XP\_065068103\_13697-model1.83 | -21.414 |
|  | XP\_065068103\_13697-model1.84 | -21.4081 |
|  | XP\_065068103\_13697-model1.85 | -21.4 |
|  | XP\_065068103\_13697-model1.86 | -21.3817 |
|  | XP\_065068103\_13697-model1.87 | -21.3814 |
|  | XP\_065068103\_13697-model1.88 | -21.3655 |
|  | XP\_065068103\_13697-model1.89 | -21.3574 |
|  | XP\_065068103\_13697-model1.90 | -21.3418 |
|  | XP\_065068103\_13697-model1.91 | -21.3268 |
|  | XP\_065068103\_13697-model1.92 | -21.3212 |
|  | XP\_065068103\_13697-model1.93 | -21.3121 |
|  | XP\_065068103\_13697-model1.94 | -21.2852 |
|  | XP\_065068103\_13697-model1.95 | -21.282 |
|  | XP\_065068103\_13697-model1.96 | -21.2781 |
| # cluster 2 - Size: 1 |  |  |
|  | XP\_065068103\_13697-model2 | -21.839 |
| # cluster 3 - Size: 1 |  |  |
|  | XP\_065068103\_13697-model3 | -19.6145 |
| # cluster 4 - Size: 1 |  |  |
|  | XP\_065068103\_13697-model4 | -17.7428 |
|  |  |  |
